# An overview of tuberculosis outbreaks reported in the years 2011–2020

**DOI:** 10.1186/s12879-023-08197-w

**Published:** 2023-04-20

**Authors:** Lidia Żukowska, Daria Zygała-Pytlos, Katarzyna Struś, Anna Zabost, Monika Kozińska, Ewa Augustynowicz-Kopeć, Jarosław Dziadek, Alina Minias

**Affiliations:** 1 Institute of Medical Biology, Polish Academy of Sciences, Lodz, Poland; 2 The Bio-Med-Chem Doctoral School of the University of Lodz and Lodz Institutes of the Polish Academy of Sciences, Lodz, Poland; 3 Institute of Biology and Biotechnology, College of Natural Sciences, University of Rzeszów, Rzeszów, Poland; 4 Department of Microbiology, National Tuberculosis and Lung Diseases Research Institute, Warsaw, Poland

**Keywords:** Tuberculosis, Tuberculosis transmission, Local outbreaks, Whole genome sequencing, Recent transmission

## Abstract

**Background:**

In many countries tuberculosis (TB) remains a highly prevalent disease and a major contributor to infectious disease mortality. The fight against TB requires surveillance of the population of strains circulating worldwide and the analysis of the prevalence of certain strains in populations. Nowadays, whole genome sequencing (WGS) allows for accurate tracking of TB transmission. Currently, there is a lack of a comprehensive summary of the characteristics of TB outbreaks.

**Methods:**

We systematically analyzed studies reporting TB outbreaks worldwide, monitored through WGS of *Mycobacterium tuberculosis*. We 1) mapped the reported outbreaks from 2011- 2020, 2) estimated the average size of the outbreaks, 3) indicated genetic lineages causing the outbreaks, and 4) determined drug-resistance patterns of *M. tuberculosis* strains involved in the outbreaks.

**Results:**

Most data originated from Europe, Asia, and North America. We found that TB outbreaks were reported throughout the globe, on all continents, and in countries with both high and low incidences. The detected outbreaks contained a median of five *M. tuberculosis* isolates. Most strains causing the outbreaks belonged to lineage four, more rarely to lineage two. Reported outbreak isolates were often drug resistant.

**Conclusions:**

We conclude that more WGS surveillance of *M. tuberculosis* outbreaks is needed. Globally standardized procedures might improve the control of *M. tuberculosis* infections.

**Supplementary Information:**

The online version contains supplementary material available at 10.1186/s12879-023-08197-w.

## Background

Tuberculosis (TB) is a communicable infectious disease caused by mycobacterium species belonging to the *Mycobacterium tuberculosis* complex. In most cases, the disease affects the respiratory system of the lung. However, TB can also involve lymph nodes, the central nervous system, bones, and joints and take a disseminated form [1, 2]. For several years, a decrease in TB cases can be observed worldwide, but the number of people contracting TB each year is still very high. In 2019, 10 million people contracted TB, and 1.2 million died from it, keeping TB among the top causes of death from infectious diseases alongside HIV/AIDS and, nowadays, COVID-19 [3]. The COVID-19 pandemic has slowed and even halted progress against TB. Complete eradication of TB worldwide seems difficult for various reasons. Currently there is no known therapy to completely eliminate latent TB. To date, there is no efficient vaccine giving adults herd immunity. The treatment of TB is difficult and consists of at least 6 months of drug administration. Drug-resistant strains are increasing – there were 160 684 drug-resistant TB cases in 2017, 186 772 in 2018, and 206 030 in 2019 [4–6].

Since so many people get affected by TB, often carrying high costs of treatment and facing poverty because of it, various agencies like World Health Organization (WHO), Centers for Disease Control and Prevention (CDC) and European Centre for Disease Prevention and Control (ECDC) implemented special programs to help combat TB. Breaking the chain of transmission is one of the most critical targets in TB eradication [7].

Before applying genetic methods in epidemiological investigations, *M. tuberculosis* transmission was monitored through the differentiation of strains based on phenotype. However, this method is not very practical for slow-growing organisms and is used only for drug resistance testing. Thanks to developments in molecular biology, genetic typing through repetitive or non-repetitive sequences and SNPs can be used. The first methods include IS*6110*, spoligotyping, and MIRU-VNTR, of which the last two are the most used in laboratory practice, especially in developing countries. IS*6110* is based on RFLP analysis coupled with identification of the site of insertion. MIRU-VNTR is based on the identification of variation in polymorphic tandem repeats in specific chromosome regions. Spoligotyping is a PCR-based method analyzing the structure of the CRISPR-Cas *locus*. Discrimination occurs by analyzing the presence or absence of spacers in the 43-spacer set [8]. It is generally accepted that methods like spoligotyping or MIRU-VNTR can exclude transmission but are not sufficiently discriminative to confirm transmission chains. This is because genetic markers indicated by each method may show identical results for distinct strains due to convergent evolution and the lack of resolution needed to detect recent transmission [9]. Transmission chain confirmation is also done through whole-genome sequencing (WGS) and/or epidemiological/contact investigations. The advantage of WGS is its high discriminatory power since it analyzes vast genome regions. Furthermore, homoplasy and backward mutations are rare, and WGS mainly relies on SNPs (28). Additionally, the precision of data obtained through WGS allows for advancements in the mathematical modeling of transmission and machine learning [10]. Importantly, conventional genotyping is critical in current epidemiology, it is widely used, and it often precedes whole genome sequencing, especially for outbreak investigation.

Based on single nucleotide polymorphisms (SNPs) identification in other than drug resistance-associated genes, the global population of *M. tuberculosis* can be phylogenetically divided into nine major lineages, distributed in a way associated with specific geographic regions [11] due to tight association with historical human migration patterns [12]. Based on the presence of the TbD1 region (MmpS6/MmpL6-encoding Mtb-specific deletion region), *M. tuberculosis* lineages are divided into ancient and modern [13]. The modern lineages are mostly Eurasian lineages like lineage two (L2) (East Asian), L3 (East-African-Indian), and L4 (Euro-American). The ancient lineages are L1 (Indo-Oceanic) and the most restricted, only to specific regions of Africa, lineages L5 (West African 1), L6 (West African 2), and L7 (Ethiopia) [12]. Lineages 5 and 6 are also referred to as *M. africanum* [14]. Besides these seven lineages, two new lineages, L8 and L9, were separated from *M. africanum* based on phylogenomic analyses, drug resistance mutations and geographical distribution [15, 16]. Modern lineages are generally regarded as more worldwide spread than ancestral ones, which are considered more endemic [17, 18]. L2 is found mainly in East and Central Asia, while L4 is identified mainly in Europe, America, and Africa; both are found worldwide. L1 is found mainly in East Africa, the Philippines, and L7 in Ethiopia [12]. L4 has very high genetic diversity compared to other lineages and includes different sublineages with different geographic distribution e.g. X (4.1.1), Haarlem (4.1.2), Ghana (4.1.3), Cameroon (L4.6.2), Uganda (L4.6.1), LAM (4.3) [19]. L2 is compromised out of 2 sublineages, proto-Beijing or ancient Beijing (2.1) and modern Beijing (2.2), which is much more diverse than the ancient Beijing sublineage [20].

Currently, there is a lack of a comprehensive summary of the characteristics of TB outbreaks. The objective of this study was to synthesize available information on *M. tuberculosis* strains involved in the outbreaks to 1) map the reported outbreaks from 2011- 2020, 2) estimate the size of the outbreaks, 3) indicate genetic lineages causing the outbreaks, and 4) determine drug-resistance patterns of *M. tuberculosis* strains involved in the outbreaks. We focused on studies where the WGS of strains confirmed local outbreaks.

## Materials and methods

The research aimed to summarize four characteristics of tuberculosis outbreaks: 1) the location of the outbreaks, 2) the size of the outbreaks, 3) the phylogenetic background of outbreak strains, and 4) the drug resistance pattern of outbreak strains. We systematically screened peer-reviewed reports deposited in the Pubmed database and Web of Science database for TB outbreaks confirmed by the currently most reliable epidemiological method, WGS.

### Definitions and constraints

#### A cluster

A cluster was defined as two or more strains linked genetically through WGS.

#### An outbreak

An outbreak was defined as at least three cases of infection with evidence of serial transmission or a cluster consisting of at least three strains, as used previously [21–23].

#### Differentiation of population-based, drug-resistant population-based and outbreak investigation studies

Population-based studies were characterized by the use of large numbers of strains usually collected in National Reference Centers or Research Facilities to indicate strain population structure from more extensive areas. Drug-resistant population-based studies included strains from large collections but focused on drug-resistant strains population. Outbreak investigations concerned strains collected and spread in a specific place, sharing the same genotyping pattern obtained by classical methods of differentiation- spoligotyping and/or MIRU typing.

#### Time constraints on the indication of transmission

Since *M. tuberculosis* may enter a state of latency for decades, local transmission of clonal strains may be detected over long periods if the outbreak contains many isolates. All analyzed strains in this study were isolated within the last 30 years, which is within the lifespan of a single host. We did not impose time-range criteria on local outbreak studies included in this review. Of note, following the assumption that the mutation rate of *M. tuberculosis* is 0.3 SNP per year [24], the restriction of ≤ 12 SNPs to define recent transmission restricts transmission prior to approximately 20 years.

#### Location constraints on the indication of transmission

We classified the studies with five distinct levels of local outbreak extent: exact location (for example, a particular building), city, region, country, or international. We included international surveillance studies due to the current significant level of globalization and international travel.

#### Strain-relatedness constraints on the indication of transmission

We focused on studies where transmission cluster data based on WGS followed the rule, where two isolates involved in an outbreak differed no more than by 12 SNPs, indicating recent transmission. If there were no such criteria, but it was possible to extract data, we used the ≤ 12 SNPs rule to identify outbreak clusters.

#### Drug resistance

We reported data on drug resistance where available. A drug-resistant variant was defined when it was resistant to at least one first-line antibiotic used in the treatment of TB. There was no restriction on the identification method of drug resistance, whether it was reported through the in silico analysis of mutations or phenotypic testing.

### Search strategy and inclusion/exclusion of articles

We conducted a systematic literature search of TB outbreaks worldwide identified by WGS. The search strategy was based on searching two major electronic databases, the PubMed and the Web of Science. The timeline restriction was 25 Nov 2020. We searched for phrases "whole genome sequencing tuberculosis" and "tuberculosis outbreak". We used the COVIDENCE tool (Covidence, Australia), the standard production platform for Cochrane Reviews, for screening and data collection.

Three authors, LZ, DZ, and AM, screened the articles. The articles obtained from the database search were screened in two steps: abstract and title screen, followed by a full-text screen and data extraction. At least two authors screened each manuscript, and in case of discrepancies, the consensus was reached by at least three authors. We considered English written manuscripts referencing human tuberculosis, where clustering of strains was determined by WGS (Table 1).

**Table 1:** Criteria for including and excluding articles in this study

Including	Excluding
*M. tuberculosis* studies	Other species of *Mycobacteria*
Human TB	Animal TB or zoonotic transmission of TB
Whole genome sequencing of at least three *M. tuberculosis* isolates from distinct people	Less than three isolates from distinct people sequenced and studies designed to investigate isolates from less than three possibly epidemiologically-linked people
Clustering analysis based on 12 or less SNP rule for clustering or possibility to identify clusters of 12 or less SNP of difference	Lack of clustering analysis or clustering analysis based on strains differing by more than 12 SNP, impossible to impose 12 or less rule for clustering
Cluster after sequencing containing three or more isolates	Largest cluster after sequencing containing less than three isolates or impossible to determine clusters of three and more isolates
English language	Any other language than English
Original analysis	Repurposed data or review manuscripts
	Study not available in full text

### Statistics

The descriptive statistics of median and interquartile range used in this manuscript were calculated with Excel Microsoft Office (Microsoft, USA).

## Results

### Literature screening

**Fig. 1:**
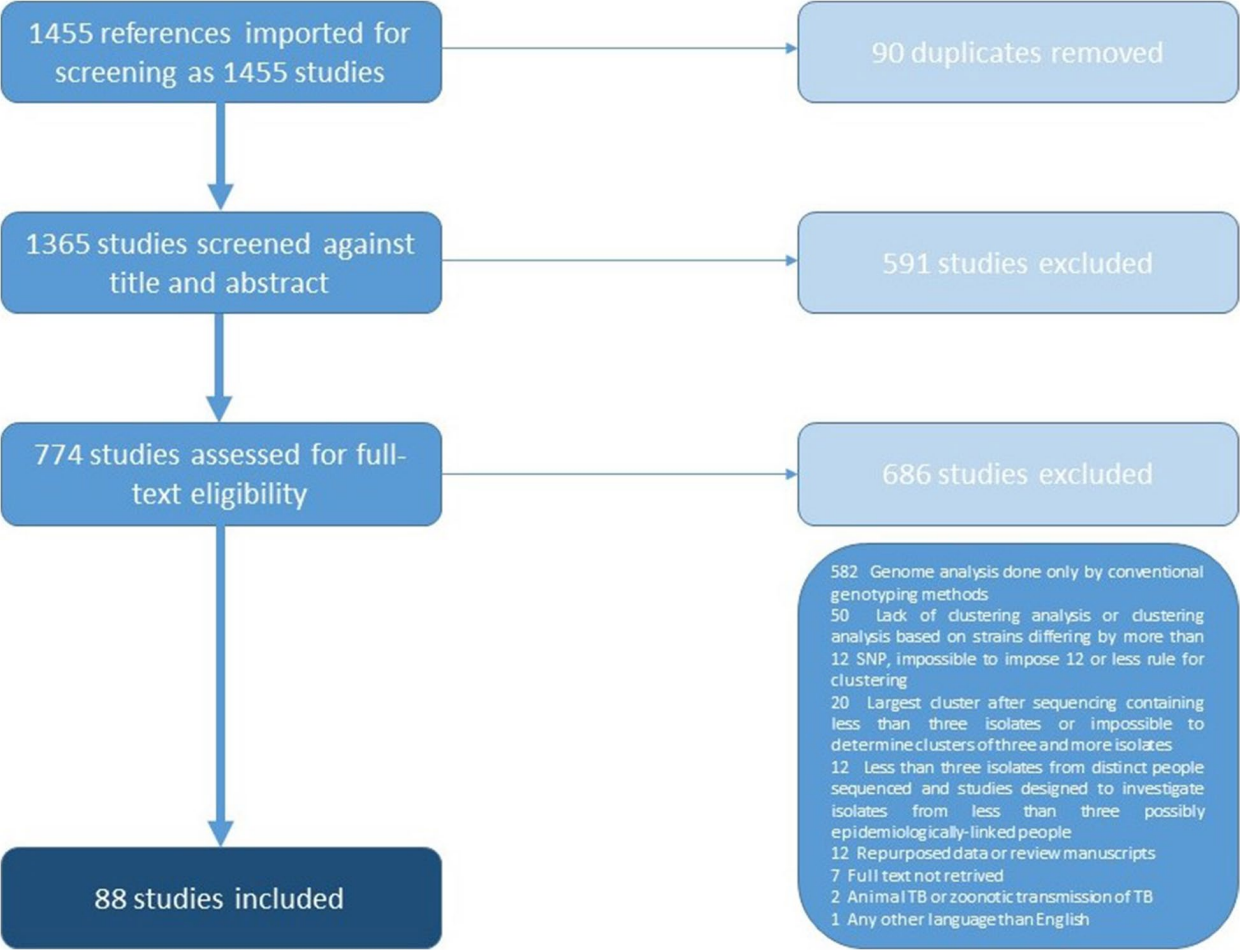
The PRISMA overview of the process of obtaining data for analysis. We used the COVIDENCE tool to analyze data efficiently. We searched PubMed and Web of Science databases for the phrases "whole genome sequencing tuberculosis" and "tuberculosis outbreak" restricted to 25 ^Nov^ 2020. Each article was screened by at least two authors based on the title and abstract. Subsequently, articles that seemed to match the criteria were screened in full. We excluded studies written in languages other than English, describing other species of mycobacteria, and repeating data from previous years. We restricted articles that referred to human TB. The inclusion criteria were confirmation of transmission thru WGS

One thousand three hundred sixty-five studies, excluding duplicates, went through title and abstract screening by at least two authors. In case of conflict between the authors, a consensus was reached. The vast majority of articles were found irrelevant to the topic of this review because they were either review articles or did not meet inclusion/exclusion criteria. Eighty-eight articles were chosen for data extraction through COVIDENCE (Fig. 1. Table S1).

**Fig. 2:**
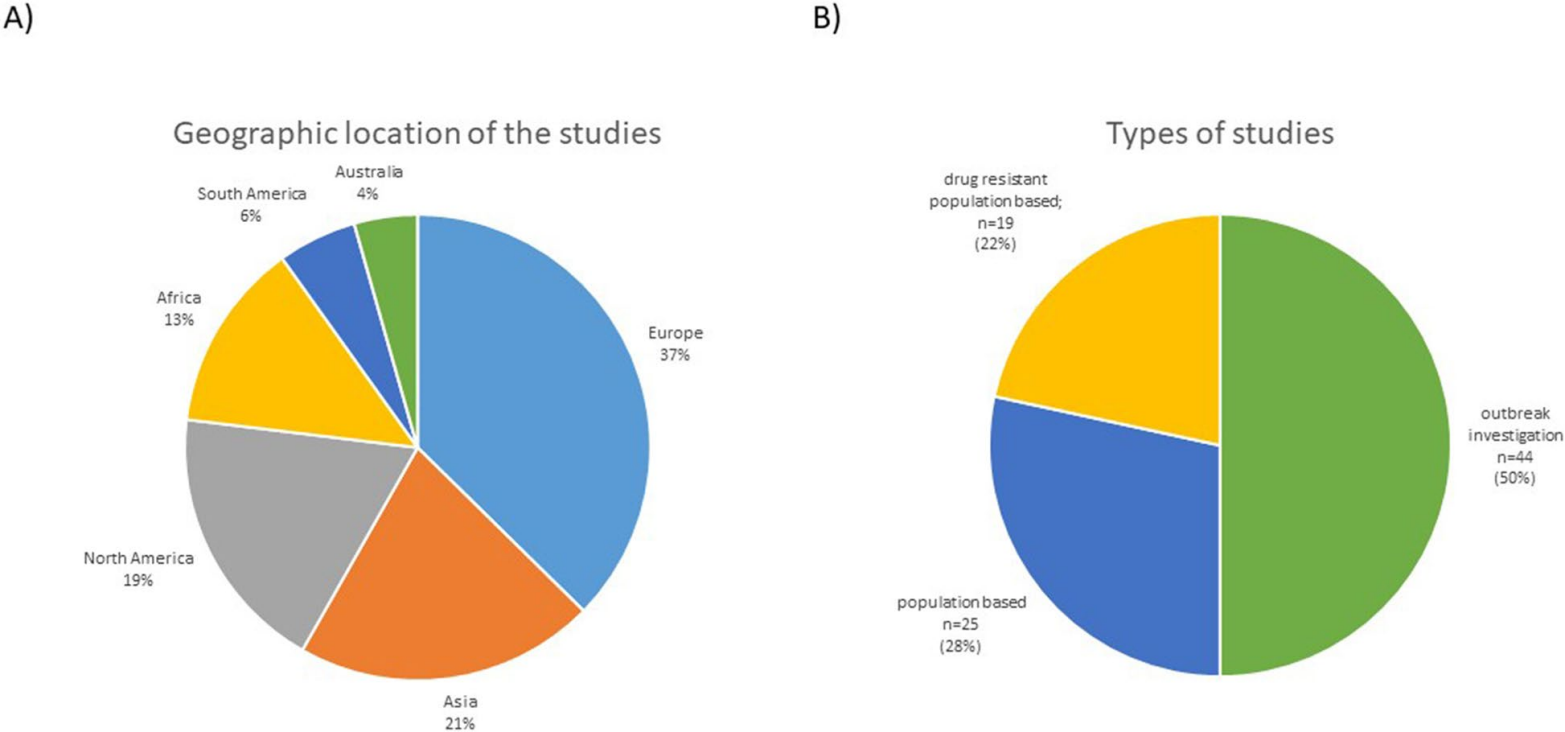
The charts summarizing article data. **A** Geographic location of studies included in our dataset. Most studies that matched our criteria concerned Asia, North America, and Europe. Africa, Australia, and South America were less represented in our dataset. **B** Types of studies included in our dataset. Half of the studies were written in the form of the outbreak investigation, where strain relatedness was suspected or detected by traditional genotyping methods, like spoligotyping and MIRU-VNTR

Data was dominated with reports mainly concerning Europe and Asia with 37% (*n* = 34) and 21% (*n* = 19) for each continent, respectively (Fig. 2A). For other continents, the number of reports was slightly smaller- North America 19% (*n* = 17) and Africa 13% (*n* = 12). The least represented were South America, 6% (*n* = 5), and Australia, 4% (*n* = 4).

The dataset included three types of studies (Fig. 2B). Two kinds of population-based studies assessed all strains in the population and drug-resistant strains in the population. The third type of study was "outbreak investigations," representing half of the reports included in our analysis. The outbreak investigations spanned over median 5 years (IQR 2–9.75).

### The geographic location of outbreaks

**Fig. 3:**
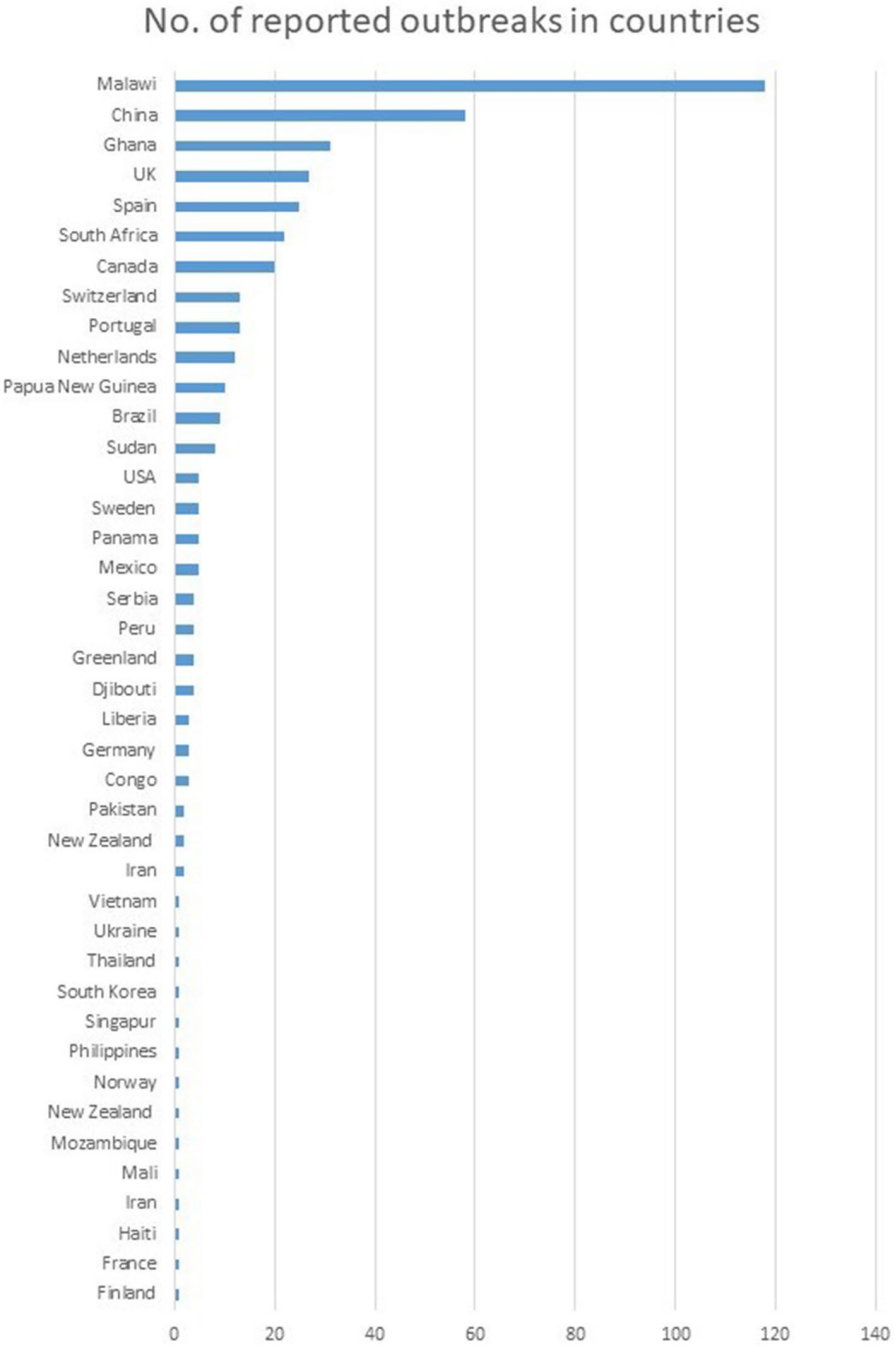
The geographic location of outbreaks. We screened the set of articles and excluded data regarding strain relatedness based on the assumption that no more than 12 SNPs separate strains involved in outbreaks. We counted the number of outbreaks reported for each country. We found outbreaks were reported by high and low-incidence countries

Outbreaks were reported from countries located on all six continents included in our analysis (Fig. 3). Most outbreaks were reported in Malawi (*n* = 118), China (*n* = 58), and Ghana (*n* = 31). The notable number of outbreaks was also reported in European countries- United Kingdom, and Spain (27, and 25, respectively).

### Size of the outbreaks

**Fig. 4:**
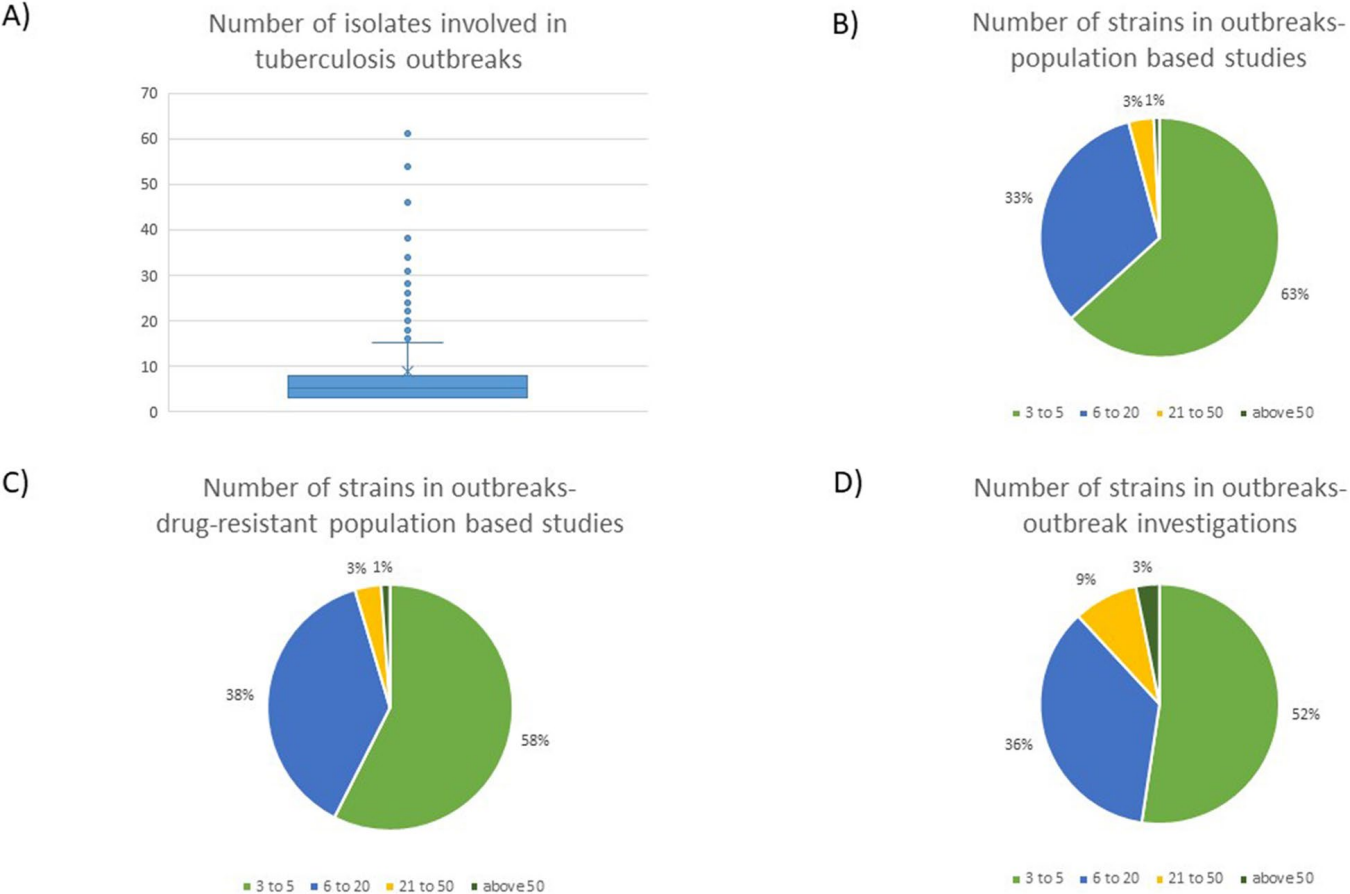
The number of isolates per each reported outbreak. We retrieved information regarding the size for 477 outbreaks. **A** Outbreak size for all analyzed studies. Five outbreaks, exceeding 65 isolates are not shown on the graph for better median and interquartile range visibility. The median number of *M. tuberculosis* isolates involved in an outbreak was 5 (IQR 3–8). **B**,** C**,** D** Outbreak size for population based studies, drug-resistant population based studies and outbreak investigations. Outbreak investigations revealed slightly bigger size outbreaks than the other two types of studies

Across the 477 outbreaks for which we were able to extract data, the median number of *M. tuberculosis* isolates involved in an outbreak was five (IQR 3–8). Outbreak investigation studies revealed slightly bigger size outbreaks than the other two types of studies (Fig. 4). There were two reports of massive outbreaks which included more than a hundred isolates- in Greenland [25], and London, United Kingdom [26].

Greenland is one of the world's least populated and relatively isolated regions. The authors sequenced *M. tuberculosis* isolates from all culture-positive cases gathered in East Greenland over 21 years (*n* = 182). The authors clustered isolates into four major clusters. The biggest cluster was estimated to evolve around 1972 in Tasiilaq and surrounding locations. The vast majority of isolates, 122, differed with ≤ 12 SNPs. The other two notable clusters were genetically similar and centered in different locations. The authors speculated that the most recent common ancestor of the three clusters was introduced in Greenland in 1894 during the foundation of a Danish colony in Tasiilaq. In summary, the study reported a very low diversity of circulating *M. tuberculosis* strains in East Greenland, with the outbreak possibly linked to "the founder effect" event over a hundred years ago.

In London, the authors sequenced 344 isolates collected over 14 years that were previously linked to an outbreak with traditional typing methods- IS6110 typing, spoligotyping, and MIRu-VNTR typing. The maximum number of SNPs between any pair of isolates was nine. The epidemiological investigation showed the outbreak spread in prison and squat frequented by intravenous drug abusers.

### The lineage of strains involved in outbreaks

**Fig. 5:**
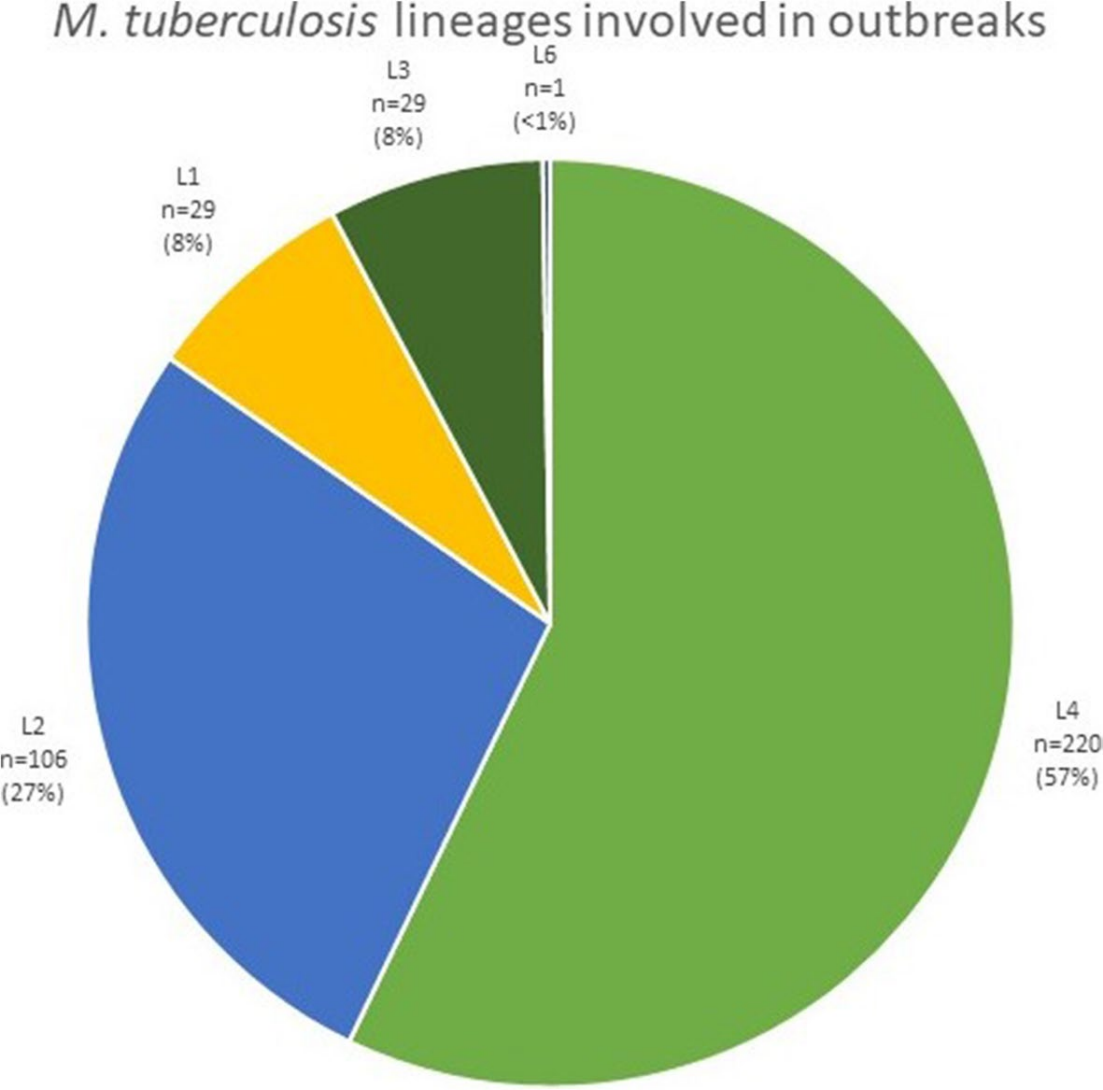
The phylogenetic lineage of *M. tuberculosis* strains involved in outbreaks. Data about lineage was available for 385 outbreaks. We did not find reports of outbreaks of L5 and L7-9

Data about lineage was available for 385 outbreaks. The most frequently reported lineage in outbreaks strains was L4 (57%, *n* = 220) (Fig. 5). The second most frequent lineage was L2, (27%, *n* = 106). We also observed minor numbers of outbreaks caused by lineages 1, 3, and 6, with L6 represented by one outbreak. The transmission of L6 took place in Ghana. It was not confirmed the transmission was not human to human [27].

### Drug resistance of outbreak-associated strains

**Fig. 6:**
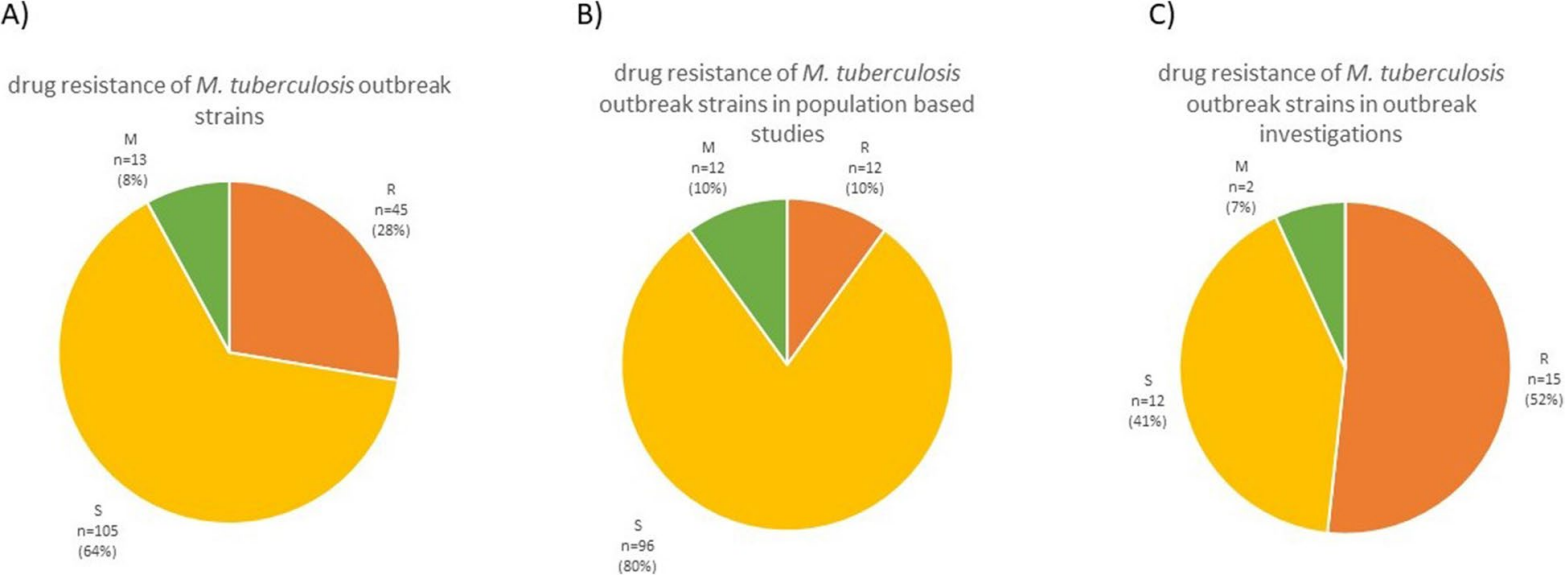
The overview of drug resistance patterns of isolates reported in outbreaks. **A** overview of *M. tuberculosis* outbreak strains drug resistance **B**) isolates in population-based studies, and **C**) isolates in outbreak investigations. The outbreak was considered drug resistant if at least any strain in the cluster was drug resistant

Information on drug resistance in particular outbreaks was available for 163 outbreaks (Fig. 6). Pansusceptible outbreaks were the most prevalent ones, constituting 64% (*n* = 105) of the total. Drug-resistant outbreaks appeared in 28% of cases (*n* = 45). Mixed outbreaks, where drug resistance was acquired over time, were the smallest fraction of the total (8%, *n* = 13). Drug resistance was notably more often reported in outbreak investigations (52%) than in population based studies (10%) (Fig. 6B, and C).

## Discussion

### Study overview

We found that TB outbreaks are reported globally, on all continents, in both high and low-incidence countries. The detected outbreaks usually contain around five *M. tuberculosis* isolates. The strains causing the outbreaks typically belong to L4, more rarely to L2. Reported outbreak isolates often are or become drug resistant.

Many factors contribute to the transmission of TB. The immune competence of the host is an important facet because it affects whether TB develops into a primary infection or turns into a latent TB [28]. The rate at which a person produces droplets containing bacteria is also important [29]. For unclear reasons, gender is also an important determinant of acquiring TB: TB seems to affect men two times more than women worldwide [30]. In 2019, 56% of people who contracted TB were men (aged ≥ 15 years) [4]. There is also rising evidence that some people are superspreaders, which means they are more infectious than others [31, 32]. Other significant factors are co-infections and coexisting diseases impacting immunity, like diabetes, alcoholism, and smoking. Apart from host-related factors, different environmental factors also play important roles in TB transmission, e.g., duration of time spent in close proximity of the infected person or country's medical infrastructure, signifying faster treatment and detection of infected people [29].

It is not well known to what extent pathogen factors contribute to the development and spread of TB since mycobacteria lack typical virulence factors [17]. The virulence level does not necessarily go along with the increased transmission. Our results support that certain genetic lineages of *M. tuberculosis* are more adapted to spread and cause disease in humans. This review's results agree with observation reporting widespread lineages two and four [33]. It is unclear whether these particular lineages are more adapted to the infection process. However, L2 and L4 seem to spread more and are often associated with increased virulence or high transmission [34]. There is possibility that certain lineages, sublineages, or strains within the sublineage, may have intrinsic genes increasing their resistance and subsequently transmission, like the Cameroon genotype [35]. The Beijing lineage shows significant strain characteristics variability, including transmissible and low-transmissible strains [36]. Compared with L4 and L2, L1 and L3 are much more geographically restricted [], which is reflected in this review. L5 seems less transmissible, possibly causing outbreaks thru infection from the external source [37]. Similar observations were made for L6 [38]. L8 is highly restricted and found only in the African Great Lakes region, but it was so far identified only in 2 patients [16]. Similarly, L9 was identified only in a few patients [15].

We observed the highest number of outbreaks in high-burden countries- Malawi, Ghana, and China. However, the high number of reported outbreaks is also likely a result of the technological availability of WGS technology, for a significant number of outbreaks was also reported in low-incidence countries of the European region.

The summarized results show that 36% of outbreak reports involved at least one drug-resistant isolate. According to WHO data, rifampin-resistant strains constitute less than 7% of the global population [5]. It seems reasonable to assume that drug-resistant outbreaks gather more attention than drug-susceptible ones, probably resulting in more frequent sequencing of such strains. Indeed, drug resistance was notably lower in population based studies than in outbreak investigations. However, based on the results summarized in this review, it should be monitored carefully whether drug-resistant strains are more likely to transmit and cause disease. In early studies, MDR/XDR strains were thought to be less virulent in a guinea pig model, which meant they were less adaptable to the environment of a new host (not likely to spread); therefore, they were less evolutionary fit [39]. For that reason, there was less concern than should have been for MDR strains causing outbreaks. While some drug-resistant strains may be less fit, they constitute a significant percentage of all circulating *M. tuberculosis* strains.

### Study limitations

It needs to be stressed that the data obtained in this review is possibly biased. Strains in which drug resistance is detected tend to receive more attention than the susceptible strains. Often, laboratories genotype only certain strains due to money restrictions, which usually means sequencing only MDR or XDR strains. Therefore outbreak strains reported in this review do not necessarily represent the *M. tuberculosis* population. Another possible problem is that there were reports that included longitudinal samples together with isolates obtained from distinct patients. Such an approach may have skewed the results regarding the outbreak frequency. Finally the actual size of the outbreaks, especially for outbreak investigations, may be skewed. Conventional genotyping methods usually detect larger groups of possibly linked cases, yet only a fraction of isolates undergoes WGS.

### Recommendations for reporting outbreak studies

A very small fraction of isolates is currently being sequenced. If we are to control the TB epidemic, we need more information regarding transmission chains. Identification of exact routes of transmission could facilitate the identification of superspreaders and asymptomatic patients. Even though it is not currently achievable to introduce WGS to the routine diagnosis of TB on a global scale, we should encourage those types of studies to increase our control over the epidemic. The more strains we sequence, the more we will know about the characteristics of the global population of *M. tuberculosis* and its ability to transmit and cause disease.

Collecting data about outbreaks should be precise and standardized to facilitate data extraction. We recommend that for efficient reporting of outbreaks, authors should catalog analyzed strains in DNA sequence repositories, for example European Nucleotide Archive or NCBI Genome Database. The information about strains should include information about the date and place of collection and the drug resistance of the strain. Preferably, patient data should also be included. The information about outbreaks should include the starting dates in which the outbreak is observed, the number of patients affected by the outbreak, and the area where the outbreak was detected. Further, the authors should state the rules conferring genetic relatedness between the two strains and precise data about strain lineage.

## Conclusions

We conclude that TB outbreaks are reported globally, contain median of five isolates that typically belong to L4, and often are or become drug resistant. Our study summarizes data available up to date and indicates areas of research that require organization and future development.

### Supplementary Information

**Additional file 1.**

## Data Availability

All data was collected from publically published manuscripts. All datasets were referenced in the supplementary material of this manuscript.

## Abbreviations

CDC: Centers for Disease Control and Prevention
ECDC: European Centre for Disease Prevention and Control
L1–9: Lineage one – Lineage nine
MDR: Multidrug – resistant
R: Resistant
RR: Rifampicin monoresistant
SNPs: Single nucleotide polymorphisms
TB: Tuberculosis
WGS: Whole genome sequencing
WHO: World Health Organization
XDR: Extensively drug – resistant strains
