# Correction: An overview of Tuberculosis outbreaks reported in the years 2011–2020

**DOI:** 10.1186/s12879-023-08751-6

**Published:** 2023-11-02

**Authors:** Lidia Żukowska, Daria Zygała-Pytlos, Katarzyna Struś, Anna Zabost, Monika Kozińska, Ewa Augustynowicz-Kopeć, Jarosław Dziadek, Alina Minias

**Affiliations:** 1 Institute of Medical Biology, Polish Academy of Sciences, Lodz, Poland; 2 The Bio-Med-Chem Doctoral School of the University of Lodz and Lodz Institutes of the Polish Academy of Sciences, Lodz, Poland; 3 Institute of Biology and Biotechnology, College of Natural Sciences, University of Rzesz?w, Rzesz?w, Poland; 4 Department of Microbiology, National Tuberculosis and Lung Diseases Research Institute, Warsaw, Poland

**BMC Infectious Diseases (2023) 23:253**

10.1186/s12879-023-08197-w

The original publication of this article contained an incorrect funding number, the incorrect and correct information are available in this correction article. The original article has been updated.

Incorrect

This work is a part of the research project financed by the National Science Center of Poland, grant number 2019/34/E/NZ6/00221.

Correct

This work is a part of the research project financed by the National Science Center of Poland, grant number 2019/35/B/NZ7/00942.

